# Metagenomic analysis reveals taxonomic and functional diversity of microbial communities on the deteriorated wall paintings of Qinling Tomb in the Southern Tang Dynasty, China

**DOI:** 10.1186/s12866-023-02887-w

**Published:** 2023-05-19

**Authors:** Wei Xing, Binjie Qi, Rulong Chen, Wenjun Ding, Fang Zhang

**Affiliations:** Laboratory of Environment and Health, College of Life Sciences, University of Chinese Academy of Sciences, 19A Yuquan Road, 100049 Beijing, China

**Keywords:** Ancient chinese Tomb, Mural painting, Whole metagenome sequencing, Microbial composition, Functional prediction

## Abstract

The microbial colonization on ancient murals attracts more and more attention since the threaten by microorganisms was first reported in Lascaux, Spain. However, the biodeterioration or biodegradation of mural paintings resulted by microorganisms is not clear yet. Especially the biological function of microbial communities in different conditions remained largely unaddressed. The two mausoleums of the Southern Tang Dynasty are the largest group of emperor mausoleums during the Five Dynasties and Ten Kingdoms period in China, which are of great significance to the study of the architecture, imperial mausoleum systems and art in the Tang and Song Dynasties. To make clear the species composition and metabolic functions of different microbial communities (MID and BK), we analyzed the samples from the wall paintings in one of the two mausoleums of the Southern Tang Dynasty with metagenomics method. The result showed totally 55 phyla and 1729 genera were detected in the mural paintings. The two microbial community structure were similar with the dominance of Proteobacteria, Actinobacteria and Cyanobacteria. However, the species abundance presented a significant difference between two communities at genus level --- MID is Lysobacter, Luteimonas are predominant in MID while Sphingomonas and Streptomyces are popular in BK, which is partially attributed to the different substrate materials of murals. As a result, the two communities presented the different metabolic patterns that MID community was mainly participated in the formation of biofilm as well as the degradation of exogenous pollutants while the BK was predominantly related to the photosynthesis process and biosynthesis of secondary metabolites. Taken together, these findings indicated the effect of environmental factor on the taxonomic composition and functional diversity of the microbial populations. The installation of artificial lighting needs to be considered carefully in the future protection of cultural relics.

**Supplementary Information:**

The online version contains supplementary material available at 10.1186/s12866-023-02887-w.

## Introduction

The ancient murals are precious treasures in human historical and cultural heritages, which are important in the research of society, religion, architecture, and fine arts, such as the Lascaux Cave painting (France), the Altamira Cave painting (Spain), the Mogao Grottoes mural (China) and the Takamatsuzuka Tumulus mural paintings (Japan) [1–4]. However, the various diseases including crack, dissolved alkali and salt, discoloration, and microbial colonization always pose a hazard to the aesthetics and research value of murals [5–7]. Many studies have reported that microorganisms, including bacteria, fungi, archaea, algae, and lichens, are easy to colonize mural surfaces in the humid environment and result in the subsequent biodeterioration of mural paintings [8, 9]. Actinomycetes, Firmicutes, and Proteobacteria were the most dominant bacterial groups found in the ancient murals. For example, the *Stenotrophomonas* in Gamma Proteobacteria were detected in the blackish mouldy spots on a 1,300-year-old polychrome mural [10]. Proteobacteria and Actinomycetes were believed as the main cause of the yellow and gray stains on the mural of the Paleolithic period in Altamira Cave, Spain [11]. Meanwhile, the fungal communities are also detected in the mural painting, such as Pencillium, Aspergillus and Cladosporium, etc. [12]. A study on the mural paintings of St. Martins church in Germany revealed that the colored biofilms were related to the microscopic fungi with strong pigment development, such as Acremonium, Aspergillus, Cladosporium, Fusarium [5]. Moreover, the microbial community on mural paintings is usually affected by the environmental factors (substrate materials, humidity, temperature, pH and light) [13–15]. A study on the murals of a rock church in Italy showed that sunlight promoted the growth of photoautotrophic microbial communities on murals under high humidity [16]. Therefore, it is important to study the microbial composition and gene function for exploring the biodegradation of the microbial communities to the mural paintings in different environments.

With the advantage of high throughput, rapid analysis and few samples, 16 and 18 S rDNA technology or Internal Transcribed Spacer (ITS) sequencing has been widely used to analyze the species composition and diversity of bacteria and fungi in the ancient murals [17–19]. However, more information in protecting the deteriorated wall painting should be provided. Metagenomics based on the whole genome sequencing is a precise and sensitive method to reveal the composition and abundance of bacteria, fungi, and archaea simultaneously in a complex environmental samples [20]. Moreover, it can predict the gene function and metabolic pathway of the microbial community, combined with the different data banks [21, 22]. This technique has been widely applied to the studies on microbial communities in water, soil and airborne [23–25].

The Mausoleums of the Southern Tang Dynasty composed of Qinling and Shunling are the largest group of emperor mausoleums during the Five Dynasties and Ten Kingdoms period, which are of great value to the study of the architecture, imperial mausoleum systems and art in the Tang and Song Dynasties. However, due to the long history and the effect of subtropical monsoon climate, the different colored stains appeared on the wall paintings in the Qinling Tomb. So, to make clear the cause of the stains as well as its role to the murals, the whole metagenome sequencing method was applied to analyze the species composition and diversity of microbial communities in the wall paintings of the Qinling Tomb. Meanwhile, the metabolic pathway of the microbial communities was also analyzed with Kyoto encyclopedia of genes and genomes (KEGG) data bank [26].

## Materials and methods

### Sample collection

**Fig. 1:**
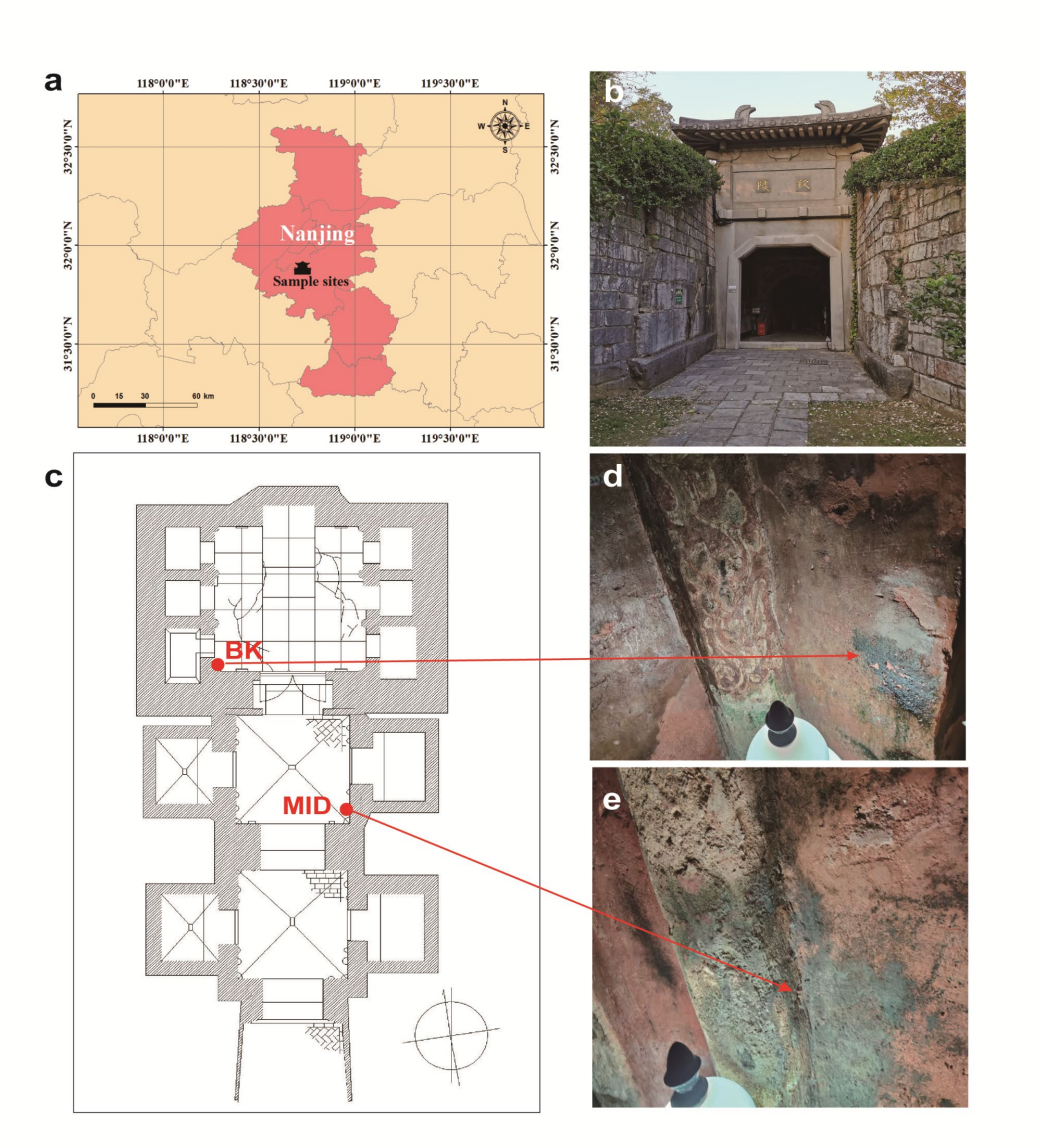
Information of the sampling sites (**a**) The geographical location of the Qinling Tomb; (**b**) The entrance of the Qinling Tomb; (**c**-**e**) The Sampling sites of MID and BK

The Qinling Tomb is located at 31°53′ North (latitude) and 118°44′ East (longitude), which lies in the southwest of Zutang Mountain of Nanjing, China. The annual average temperature is 15.4 °C with 1106 mm precipitation. The tomb includes three rooms: front room, middle room, and back room, each with side chambers. The back room and its chambers are made of limestones and the rest are made of black bricks [27]. All the walls of three rooms were painted with red decoration. As shown in Fig. 1, two sampling sites were selected in the middle and back rooms. To meet the sample size required for sequencing, six samples were collected and mixed for each site, named as MID and BK, respectively. In order to study the seasonal impact on microbial communities, three-time samplings were carried out in August 2020, November 2020, and April 2021, denoted by Aug, Nov, and Apr, respectively. The environmental temperature and humidity were measured during sampling (Table S1). All samples were collected following strictly aseptic procedures and stored in a sterile tube at -80 °C until further analysis.

### SEM analysis

The sample was placed on a sample holder to be dried at room temperature. Before analysis, the samples were coated with platinum for 90 s in a vacuum environment (I = 15 mA). The scanning electron microscopy (FEI Scanning Electron Microscope QUANTA 200FEG, USA) were applied to observe the morphology of microorganism.

### Metagenomic analysis

Genomics DNA was extracted from the samples of wall painting at each sampling site. In brief, the samples were treated with NucleoSpin Soil Kit (Macherey-Nagel, Germany) following the manufacturer’s instructions. The content of DNA was measured using Qubit dsDNA BR Assay kit (Invitrogen, USA) with a Qubit Fluorometer. The quality was checked on 1% agarose gel.

In order to prepare library construction for DNA sequencing, the genomic DNA was sheared by sonication method using Covaris (Covaris, USA). The small fragments with 200–400 bp were selected by magnetic beads. Through end-repaired, 3’ adenylated, adapters-ligation, and PCR amplifying, the products were purified by magnetic beads. The double stranded PCR products were heat denatured and circularized by the splint oligo sequence. The single strand circle DNA (ssCir DNA) were formatted as the final library and qualified by FastQC. The qualified libraries were sequenced on MGISEQ-2000 platform (BGI Shenzhen, China).

### Bioinformatics and statistical analysis

All the raw data were trimmed by SOA Pnuke v.1.5.2. High-quality reads were de novo assembled using Megahit software. Assembled contigs with length less than 300 bp were discarded in the following analysis. Genes were predicted over contigs by using Meta Gene Marker (2.10). Redundant genes were removed using CD-HIT with identity cutoff 95%. Based on the Kraken2, the taxonomic annotation was assigned and the taxonomic abundance was generated. The difference of relative abundance at the phylum level across groups were determined using Wilcoxon’s rank sum test. *P*-values for multiple testing were corrected using the BH method with corrected *P* < 0.05 were considered significant. The alpha diversity including Chao 1, Shannon-Wiener, and Simpson indexes was quantified using the relative abundance profiles at genus levels with R package. The beta diversity was calculated using Bray-Curtis distance. Principal Coordinate Analysis (PCoA) was performed by R package VEGAN. The distribution of the number of genes for each KEGG pathway was obtained based on the annotated results and the gene sets. The approach of Reporter Score was used to statistically test all the KOs involved in a pathway, and the overall cumulative trend was used to reflect the change of the pathway.

## Results

### Description of microorganisms on the wall

**Fig. 2:**
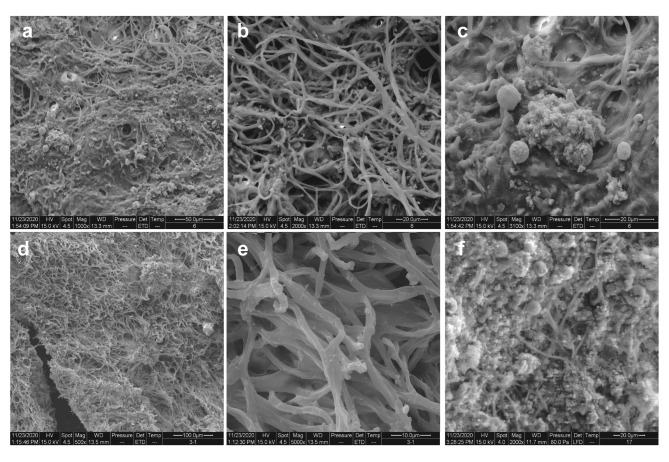
SEM images of microorganisms on the walls. The filaments and the spheroids were observed in MID (**a**-**c**) and BK (**d**-**f**)

To describe the morphology of microorganisms on the wall paintings, the samples taken from two sites were analyzed by SEM. It can be observed plenty of filaments with the diameter ranging from 2 to 4 μm and spheroids with the diameter of 5–10 μm, which are shown in Fig. 2a f.

### Microbial diversity detected on the wall paintings

**Fig. 3:**
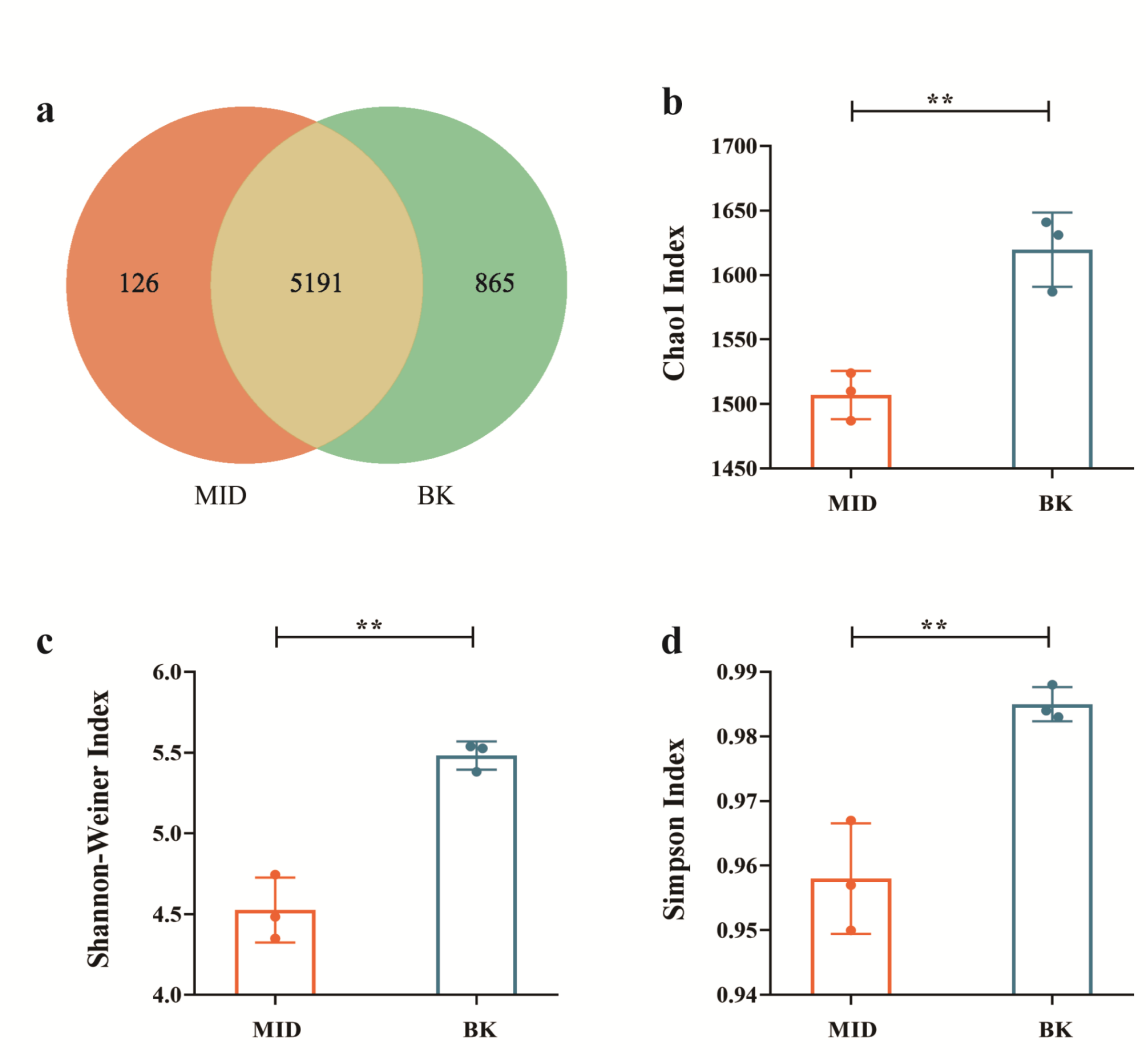
Microbial diversity of two communities. (**a**) Venn diagram of species identified in two communities; (**b**) Chao1 index; (**c**) Shannon-Wiener index. (**d**) Simpson index. ** *P* < 0.01

To know the composition of microbial communities on the wall painting, the samples of MID and BK were analyzed by whole metagenome sequencing. The summary of raw metagenome sequence data is presented in Table 1. The high-quality sequences obtained were annotated as 6182 species. By examining the database, a total of 4 kingdoms, 55 phyla, 1729 genera were detected (Table S2). Venn diagram was used to illustrate the similarity between MID and BK communities. As shown in Figs. 3a and 5191 species were shared by two sampling sites, while 126 and 865 species were specific in MID and BK, respectively.

**Table 1:** Summary of raw metagenome data for MID and BK

Sample	Sampling time	Total reads	Read length (bp)	Total data (GB)	Percent of GC content	Q20 (%)	Q30 (%)
MID	Aug	77,086,924	300	10.77	62.95	93.34	87.06
MID	Nov	79,755,886	300	11.14	63.05	95.08	89.56
MID	Apr	78,094,898	300	10.91	63.57	95.16	89.74
BK	Aug	79,427,414	300	11.10	59.78	94.45	89.07
BK	Nov	78,979,368	300	11.03	58.39	94.93	89.16
BK	Apr	78,194,716	300	10.92	60.20	95.48	90.37

The α-diversity of two communities were also assessed by the indexes of Chao 1, Shannon-Wiener, and Simpson. The Chao 1 index was used to estimate the total number of species contained in the community. The larger the value is, the more species that will be found. The Shannon-Wiener and Simpson indexes reflect evenness of different species. The larger Shannon-Wiener and Simpson indexes indicate the higher richness and distribution uniformity of the species. As shown in Fig. 3b-d, the results showed the indexes of Chao 1, Shannon-Wiener and Simpson were significantly higher in the BK, indicating the higher microbial diversity in the community of back room.

### Difference of taxonomic composition between two sampling sites

**Fig. 4:**
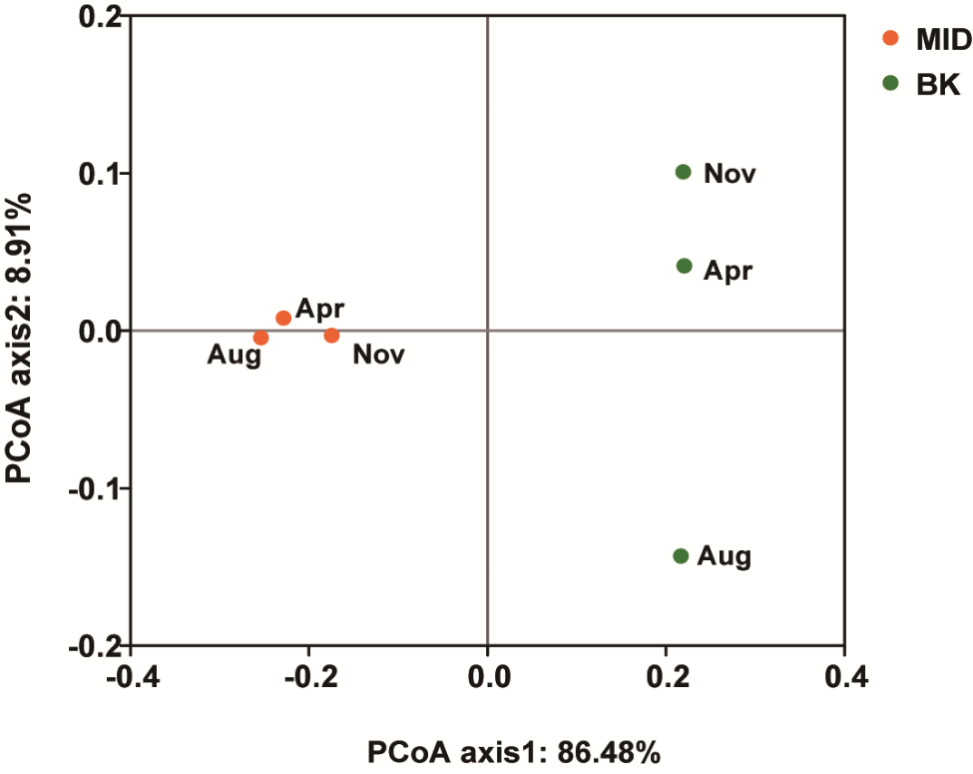
Principal coordinate analysis (PCoA) analysis diagram

The β-diversity was used to compare the similarity of microbial composition between two sampling sites by PCoA analysis. The three samples of different seasons in MID were assigned into one group according to axis1 and axis2, while the samples in BK were separated, especially the summer sample (Fig. 4). The distance according axis1 between MID group and BK group was found to be significant, suggesting a difference between two communities. Hence, the variation of composition was analyzed at different taxonomic level. From the abundance of different kingdom listed in Table S3, it was found that two communities on the mural painting were both dominated by bacteria, which accounted for up to 99%, the rest were archaea and eukaryote, the proportion of viruses was less than 0.1%.

**Fig. 5:**
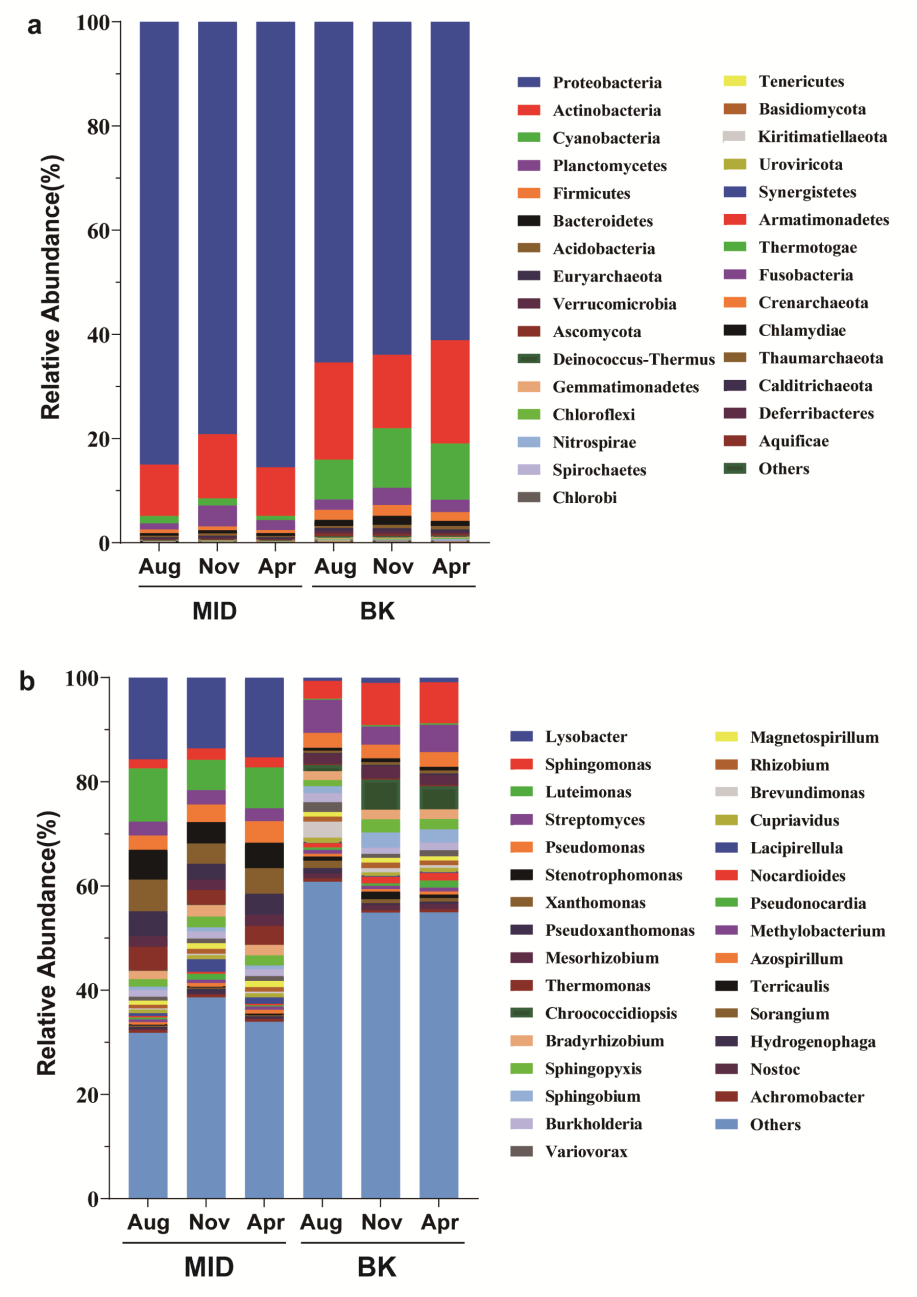
Relative abundance of the top 30 species at the phylum level (**a**) and genus level (**b**)

To make clear the difference between two communities, the abundance of top 30 species at phylum or genus level were compared respectively. As shown in Fig. 5a, the microbial composition of different samples was generally dominated by the phyla of Proteobacteria, Actinobacteria, Cyanobacteria, Planctomycetes and Firmicutes. They accounted for more than 97% of the abundance of all phyla in the sample. Proteobacteria was the most abundant group in all samples, and followed by Actinobacteria. However, the relative abundance of each phylum was varied in the different samples (Table S4). The proportion of Proteobacteria in MID is significantly higher than that in BK, but conversely, the abundance of Actinobacteria and Cyanobacteria in MID is significantly lower than that in BK (Fig. S1).

Furthermore, the abundance of species presented a significant difference between two sites at genus level (Table S5). As shown in Fig. 5b, the MID community was dominated by genera of *Lysobacter*, *Luteimonas*, *Stenotrophomonas*, *Xanthomonas*, *Pseudoxanthomonas* and *Thermotonus*, all of which were assigned to phylum Proteobacteria; while the abundance of genera *Sphingomonas*, *Streptomyces*, *Chroococcidiopsi* were obvious higher in the BK, each of which was classified to different phylum including Proteobacteria, Actinobacteria, and Cyanobacteria, respectively.

### Seasonal variation of microbial communities

**Fig. 6:**
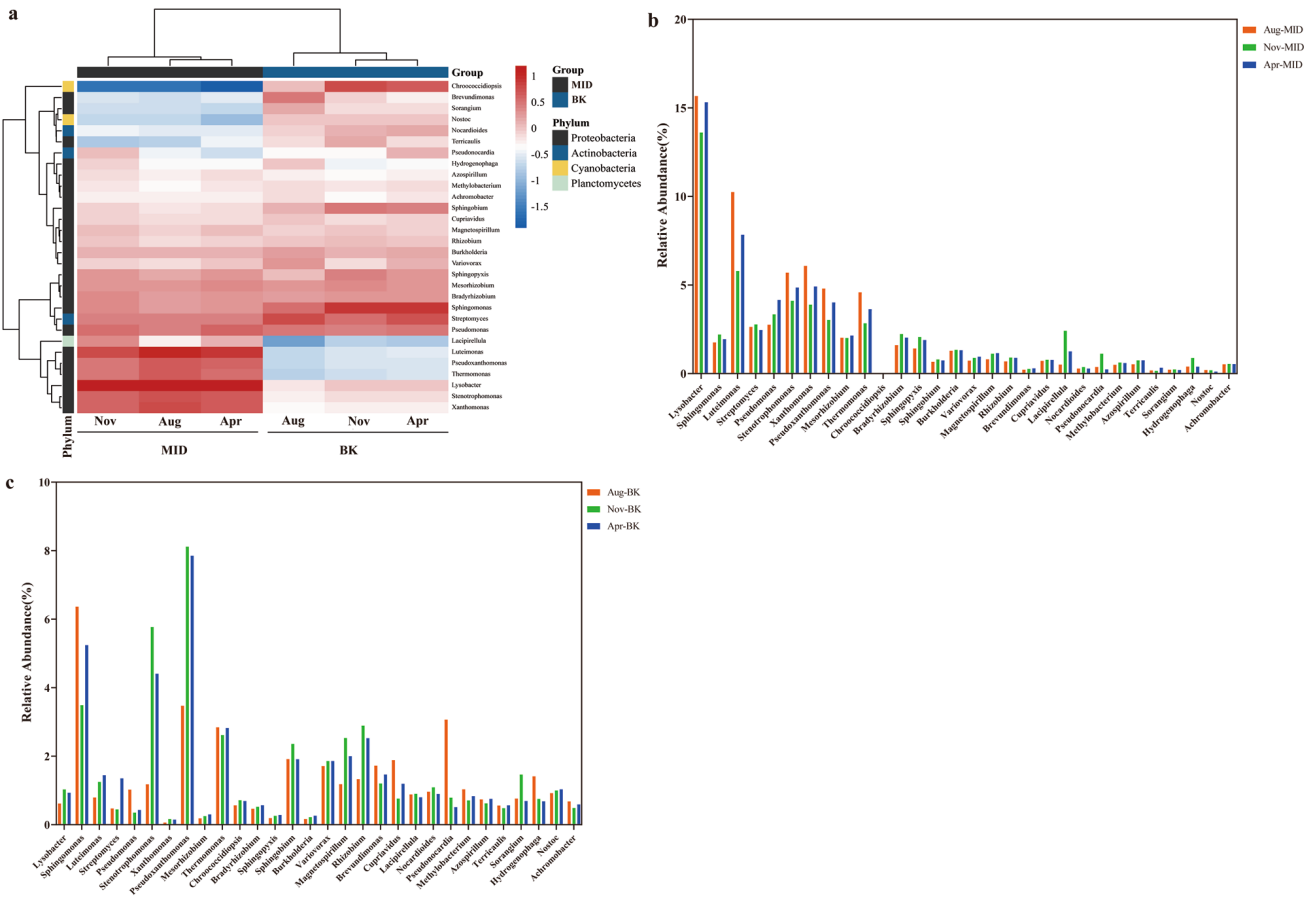
Seasonal variation of microbial communities. (**a**) Heatmap of abundance of top 30 genera in different seasons at two sites. Relative abundance of top 30 genera in MID (**b**) and BK (**c**)

To explore the seasonal impact on the microbial communities, the abundance heatmap of top 30 species at genus levels was constructed using hierarchical cluster analysis (Fig. 6a). Most of the species showed significant difference among three seasons, either in MID or BK. As shown in Fig. 6b-c, the species with high abundance including *Lysobacter*, *Luteimonas*, *Stenotrophomonas*, *Xanthomonas*, *Pseudoxanthomonas* and *Thermomonas* presented a similar season-dependent change in MID, which was least in November and most in August. But in BK, these genera had low abundance without obvious season-dependent change. Except that, most of the genera, such as *Sphingomonas*, *Chroococcidiopsis*, *Sphingopyxis*, *Sphingobium*, *Nocardioides*, had a different season-dependent variation in BK. Taken together, the seasonal impact was different on two microbial communities.

### Functional prediction of the microbial community

**Fig. 7:**
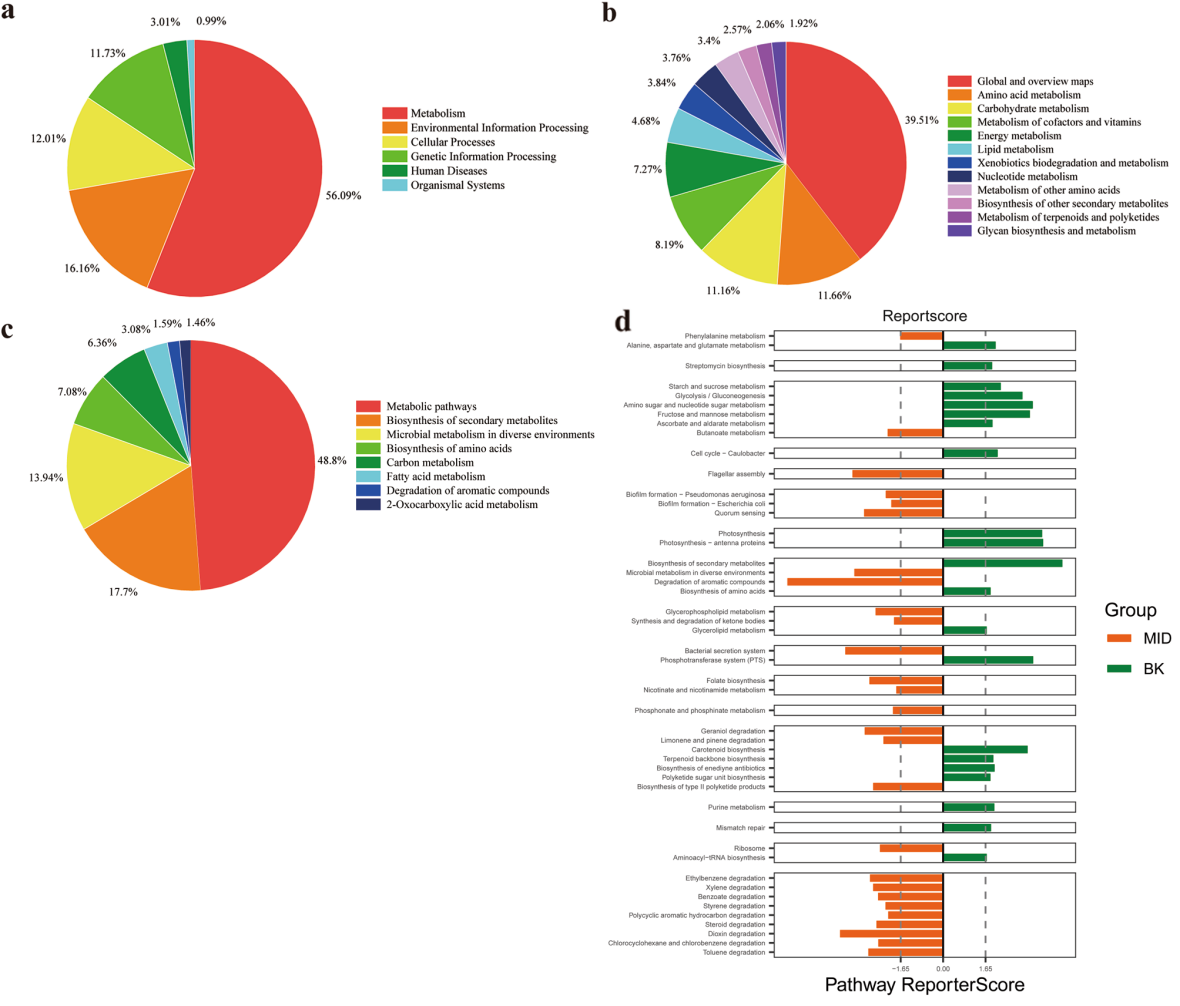
Analysis of KEGG metabolic pathway. (**a**-**c**) The percentage of pathway-related gene abundance at three levels. (**d**) The difference of metabolic pathway between two communities

To explore the potential deterioration of microorganisms to the mural paintings, the metabolic pathways of microbial communities were predicted by KEGG analysis based on metagenome data [26, 28, 29]. The biological metabolic pathway is mainly divided into three levels. At the first level (Fig. 7a), 56.09% of all identified genes were related to the metabolism, followed by the environmental information processing (16.16%), cellular processes (12.01%), genetic information processing (11.73%), human diseases (3.01%) and organismal systems (0.99%). Since most genes were involved in metabolism process, the deeper analysis at the second level for metabolism-related genes was performed. As shown in Fig. 7b, it revealed that 39.51% genes were for the metabolism of global and overview maps, followed by amino acid metabolism (11.66%), carbohydrate metabolism (11.16%), metabolism of cofactors and vitamins (8.19%), and energy metabolism (7.27%) etc. For the same reason, 17.7% genes in global and overview maps were involved in biosynthesis of secondary metabolites, followed by microbial metabolism in diverse environments (Fig. 7c). KEGG pathway results manifested the metabolic functional diversity of microbial populations on the wall painting. To compare the difference of biological function between two microbial community, the reporter score enriched by each metabolic pathway was obtained (Fig. 7d). The results indicated MID community was mainly participated in the biofilm formation and degradation of exogenous pollutants including aromatic compounds, PAHs and Dioxin, etc.; while the BK was predominantly related to the photosynthesis process and biosynthesis of secondary metabolites, such as Terpenoid, and Carotenoid. All these results manifested the metabolic diversity of microbial communities in the Qinling Tomb.

## Discussions

Many murals or wall paintings are facing serious and irreversible damage, parts of which are attributed to the microbial colonization and growth [30, 31]. While the complex ecological environment in the tomb usually leads to the microbial diversity in the murals, which results in the functional diversity of the community. In the Qinling Tomb, up to 99% identified species in the wall paintings are classified to bacteria, including phyla of Proteobacteria, Actinobacteria, Cyanobacteria, etc. This result is consistent with the previous studies on monuments [32, 33] and paintings [34, 35]. It was reported that in the different climates, Actinobacteria, Proteobacteria, Firmicutes and Cyanobacteria were the most predominant phyla within the stone-dwelling microbiomes, each playing a major ecological role in the survival of the communities on the stone [33].

However, the microbial diversity of BK community was obvious higher than that of MID. Meanwhile, the abundance of phyla Proteobacteria, Actinobacteria, and Cyanobacteria were significantly different between MID and BK, which might be related to the difference in the mural support bodies. The substrate materials of the two rooms are different——the back room is made of limestones while the middle one is made of black bricks [27]. The limestone is more appropriate for microbial development than the black brick. The microbial composition on building surfaces has been found to be highly material-specific in the same environment [36]. A previous study compared the prokaryotic and eukaryotic communities formed on mineral matrices in different geographic locations. The results showed that the substratum type was the most significant factor influencing the bacterial community [37]. Furthermore, BK contained higher abundance of Cyanobacteria (9.97%) than MID (1.21%). At genus level, Cyanobacteria in MID mainly included *Nostoc*, *Synechococcus*, *Leptolyngbya* and *Calothrix*, while BK was mainly dominated by *Chroococcidiopsis* (3.79%). All these genera are common Cyanobacteria in terrestrial habitats and colonize steles and building surfaces [38, 39]. These results indicate the microbial community is not only influenced by the environmental factors, but also by the substrate material of mural painting.

Little work was devoted to the investigations of seasonal impact on the mural microbial community. It was reported the concentration of airborne fungal propagules existed marked seasonal variation in the Dahuting Han Dynasty Tomb, higher in spring and autumn than in summer and winter [40]. In this work, we compared the variation of different community in three seasons. The results showed the diversity of community in different seasons was little varied, although the specific species were present in each season. The seasonal impact on microbial community was mainly illustrated by the abundance fluctuation of different species, for examples, *Lysobacter*, *Luteimonas*, *Nocardioides* and *Chroococcidiopsis*, etc. Temperature is an important factor affecting the growth and survival of microorganisms. It has been demonstrated the temperature sensitive microorganisms in the soils, such as phyla Actinobacteria, Proteobacteria, Planctomycetes, Bacteroidetes, and Verrucomicrobia [41]. In the Qinling Tomb, most of the season-dependent genera were also found to be phyla Proteobacteria, Actinobacteria, Cyanobacteria, and Planctomycetes. As the internal environment is relatively stable, with a perennial temperature of 14-24 °C and a relative humidity of more than 98%, the seasonal change of the mural microbial community in the Qinling Tomb is not prominent.

It has been reported a variety of biological functions of microorganisms in the environment. However, the study of the role of microorganisms in the tomb is limited. The potential function of the identified species was evaluated by KEGG analysis [26, 28]. In the Qinling Tomb, most genes of the microbial community on the wall were related to metabolic processes, such as metabolism of carbohydrate and amino acid metabolism, which are essential for microbial growth and development. A previous study showed high relative abundances of carbon metabolism and amino acid metabolism in microbial communities in caves [42]. Besides amino acids, nucleic acids, proteins and other essential substances for life, the microorganisms also synthesize and secrete a variety of secondary metabolites under stress, such as antibiotics, toxins, hormones, alkaloids, and vitamins. In the Qinling Tomb, two communities in the mural paintings presented the diverse metabolic process. As shown in Fig. 7d, the metabolic pathway related to the degradation of aromatic compounds was much prominent in the MID community, for examples, the degradation of Toluene, Chlorocyclohexane and chlorobenzene, Dioxin, Polycyclic aromatic hydrocarbon, etc. A wide variety of bacterial, fungal and algal species have the potential to degrade/transform PAHs. Alpha-proteobacteria, Beta-proteobacteria, Gamma-proteobacteria, Actinomycetes, Firmicutes and Archaea (Halophiles) are believed to have PAHs catabolic property [43]. It was reported that the PAHs degradation by bacteria mainly involved dioxygenase enzymes and partially monooxygenase mediated reactions [44]. While in the BK community, the microorganisms were mainly functioned in the biosynthesis of secondary metabolites and photosynthesis. This is probably related to the high abundant Cyanobacteria, especially Chroococcidiopsis in the BK. Cyanobacteria belongs to the phototrophic microorganism, which is usually found near the entrances or well-lit areas in the tombs or caves. Cyanobacteria can utilize atmospheric carbon to produce biomass through photosynthesis [45]. As the primary producers of the community, Cyanobacteria can provide the initial carbon sources for the stone surfaces, modify the surface characteristics and diversify the community from nutrient-poor to enrichment of heterotrophs on surfaces [46]. In the Qinling Tomb, Cyanobacteria were detected in both middle room and back room, which is partially attributed to the artificial lighting [15, 47, 48]. Therefore, the artificial illumination promoted the growth of Cyanobacteria in the Qinling Tomb. Subsequently, Cyanobacteria might provide the nutrition for the survival of heterotrophic microorganisms. As a result, the microbial community first formed in the murals near the light.

In summary, whole metagenome sequencing helps to explore the degradation of microorganisms in tomb murals, not only providing the microbial community structure like 16 or 18 S rDNA, but also predicting their biological functions. In the Qinling Tomb, the microbial community in the murals is mainly composed of bacteria, such as Proteobacteria, Actinobacteria, and Cyanobacteria, which is partially attributed to the installation of artificial lighting. Secondly, the material of the mural substrate affects the composition of the microbial community, subsequently leading to the differences in the metabolic patterns of microbial communities between the two sampling sites. However, the effect of different metabolic process of these microorganisms on mural painting needs more study. Recently, the application of multi-omics combined technology including metabolomics and proteomics is gradually applied in the study of tomb murals.

## Electronic supplementary material

Below is the link to the electronic supplementary material.

Supplementary Material 1

## Data Availability

The datasets presented in this study can be found in online repositories. The names of the repository/repositories and accession number(s) can be found below: https://www.ncbi.nlm.nih.gov/, Bioproject PRJNA904214.
