# Supplementary material for: Metagenomic analysis reveals taxonomic and functional diversity of microbial communities on the deteriorated wall paintings of Qinling Tomb in the Southern Tang Dynasty, China
Source: BMC Microbiol. 2023 May 19;23:140. doi: 10.1186/s12866-023-02887-w (PMC10197217; doi:10.1186/s12866-023-02887-w)
Supplement: Supplementary file 1 — Supplementary Material 1 [file 12866_2023_2887_MOESM1_ESM.docx]

**Supplementary Files**

**Metagenomic analysis reveals taxonomic and functional diversity of microbial communities in deteriorated wall painting of Southern Tang’s Tomb of China**

Wei Xing^†^, Binjie Qi^†^, Rulong Chen, Wenjun Ding*, Fang Zhang*

Laboratory of Environment and Health, College of Life Sciences, University of Chinese Academy of Sciences, Beijing 100049, China

**†** These authors contributed equally to this work.

* Co-corresponding authors:

Fang Zhang, PhD

E-mail: [zhangfang@ucas.ac.cn](mailto:zhangfang@ucas.ac.cn)

ORID: 0000-0003-2597-172X

Wenjun Ding, PhD

E-mail: [dingwj@ucas.ac.cn](mailto:dingwj@ucas.ac.cn)

College of Life Science, University of Chinese Academy of Sciences

19A Yuquan Road, Beijing, 100049, China

Fax: 86-10-69672641; Tel: 86-10-69672641

**Table S1** Temperature and relative humidity during sampling

| Sample | Sampling time | Temperature (℃) | Relative Humidity (%) |
| --- | --- | --- | --- |
| MID | August 2020 | 24.49±0.96 | 85.46±13.8 |
| MID | November 2020 | 16.87±1.17 | 92.42±3.04 |
| MID | April 2021 | 15.07±1.94 | 93.05±5.59 |
| BK | August 2020 | 23.77±0.39 | 99.94±0.14 |
| BK | November 2020 | 17.69±0.77 | 99.31±1.72 |
| BK | April 2021 | 15.20±1.59 | 98.77±1.83 |

Note: Data are means ± the standard error.

**Table S2** Taxonomic composition in different samples

| Sample | Sampling time | Kingdom | Phylum | Class | Order | Family | Genus | Species |
| --- | --- | --- | --- | --- | --- | --- | --- | --- |
| MID | Aug | 4 | 48 | 97 | 205 | 443 | 1510 | 5024 |
| MID | Nov | 4 | 50 | 100 | 209 | 442 | 1524 | 5064 |
| MID | Apr | 4 | 48 | 95 | 203 | 439 | 1487 | 4939 |
| BK | Aug | 4 | 54 | 101 | 211 | 459 | 1641 | 5822 |
| BK | Nov | 4 | 54 | 102 | 214 | 460 | 1631 | 5717 |
| BK | Apr | 4 | 50 | 99 | 208 | 451 | 1587 | 5517 |

**Table S3** The abundance of different kingdoms in different samples

| Sample | Sampling time | Bacteria (%) | Archaea (%) | Eukaryota (%) | Viruses (%) |
| --- | --- | --- | --- | --- | --- |
| MID | Aug | 99.530 | 0.287 | 0.159 | 0.025 |
| MID | Nov | 99.467 | 0.351 | 0.152 | 0.030 |
| MID | Apr | 99.534 | 0.299 | 0.136 | 0.030 |
| BK | Aug | 98.815 | 0.577 | 0.556 | 0.051 |
| BK | Nov | 99.052 | 0.504 | 0.407 | 0.036 |
| BK | Apr | 99.067 | 0.58 | 0.312 | 0.041 |

**Table S4** The abundance of the top 40 phyla in different samples

| Kingdom | Phylum | Abundance of various microbial phyla | | | | | |
| --- | --- | --- | --- | --- | --- | --- | --- |
|  |  | MID | | | BK | | |
|  |  | Aug | Nov | Apr | Aug | Nov | Apr |
| Bacteria | Proteobacteria | 85.010 | 79.181 | 85.516 | 65.438 | 63.924 | 61.117 |
|  | Actinobacteria | 9.860 | 12.293 | 9.305 | 18.618 | 14.105 | 19.846 |
|  | Cyanobacteria | 1.445 | 1.370 | 0.826 | 7.671 | 11.450 | 10.790 |
|  | Planctomycetes | 1.135 | 4.017 | 1.916 | 1.976 | 3.285 | 2.356 |
|  | Firmicutes | 0.707 | 0.747 | 0.603 | 1.928 | 2.083 | 1.701 |
|  | Bacteroidetes | 0.476 | 0.577 | 0.536 | 1.149 | 1.703 | 0.971 |
|  | Acidobacteria | 0.291 | 0.452 | 0.246 | 0.371 | 0.659 | 0.647 |
|  | Verrucomicrobia | 0.149 | 0.298 | 0.157 | 0.532 | 0.661 | 0.353 |
|  | Deinococcus-Thermus | 0.143 | 0.158 | 0.140 | 0.323 | 0.283 | 0.294 |
|  | Gemmatimonadetes | 0.079 | 0.109 | 0.077 | 0.164 | 0.193 | 0.203 |
|  | Chloroflexi | 0.057 | 0.085 | 0.066 | 0.150 | 0.214 | 0.187 |
|  | Nitrospirae | 0.047 | 0.049 | 0.036 | 0.098 | 0.117 | 0.310 |
|  | Spirochaetes | 0.033 | 0.025 | 0.025 | 0.067 | 0.073 | 0.066 |
|  | Chlorobi | 0.027 | 0.031 | 0.025 | 0.052 | 0.065 | 0.055 |
|  | Tenericutes | 0.005 | 0.004 | 0.003 | 0.104 | 0.034 | 0.022 |
|  | Kiritimatiellaeota | 0.015 | 0.019 | 0.016 | 0.031 | 0.039 | 0.029 |
|  | Synergistetes | 0.012 | 0.015 | 0.012 | 0.020 | 0.018 | 0.017 |
|  | Armatimonadetes | 0.007 | 0.010 | 0.007 | 0.017 | 0.023 | 0.022 |
|  | Thermotogae | 0.005 | 0.005 | 0.004 | 0.019 | 0.025 | 0.014 |
|  | Fusobacteria | 0.005 | 0.004 | 0.003 | 0.030 | 0.018 | 0.010 |
|  | Chlamydiae | 0.005 | 0.004 | 0.003 | 0.016 | 0.021 | 0.008 |
|  | Calditrichaeota | 0.004 | 0.004 | 0.004 | 0.006 | 0.008 | 0.006 |
|  | Deferribacteres | 0.003 | 0.003 | 0.002 | 0.006 | 0.008 | 0.008 |
|  | Aquificae | 0.003 | 0.003 | 0.002 | 0.007 | 0.008 | 0.004 |
|  | Chrysiogenetes | 0.004 | 0.004 | 0.003 | 0.004 | 0.006 | 0.005 |
|  | Thermodesulfobacteria | 0.002 | 0.002 | 0.002 | 0.005 | 0.006 | 0.006 |
|  | Ignavibacteriae | 0.002 | 0.001 | 0.001 | 0.005 | 0.007 | 0.005 |
|  | Balneolaeota | 0.001 | 0.002 | 0.001 | 0.003 | 0.004 | 0.005 |
|  | Candidatus_Omnitrophica | 0.002 | 0.002 | 0.002 | 0.003 | 0.003 | 0.003 |
|  | Candidatus_Bipolaricaulota | 0.001 | 0.001 | 0.001 | 0.004 | 0.003 | 0.004 |
|  | Candidatus_Saccharibacteria | 0.002 | 0.001 | 0.001 | 0.002 | 0.002 | 0.002 |
|  | Elusimicrobia | 0.000 | 0.000 | 0.000 | 0.003 | 0.003 | 0.003 |
| Eukaryota | Ascomycota | 0.140 | 0.134 | 0.120 | 0.517 | 0.372 | 0.283 |
|  | Basidiomycota | 0.019 | 0.018 | 0.017 | 0.040 | 0.035 | 0.030 |
| Archaea | Euryarchaeota | 0.279 | 0.340 | 0.290 | 0.552 | 0.478 | 0.554 |
|  | Crenarchaeota | 0.005 | 0.007 | 0.006 | 0.014 | 0.014 | 0.015 |
|  | Thaumarchaeota | 0.002 | 0.003 | 0.002 | 0.009 | 0.009 | 0.008 |
| Viruses | Uroviricota | 0.013 | 0.016 | 0.019 | 0.023 | 0.021 | 0.021 |
|  | Peploviricota | 0.002 | 0.002 | 0.002 | 0.007 | 0.002 | 0.004 |
|  | Nucleocytoviricota | 0.001 | 0.001 | 0.001 | 0.007 | 0.003 | 0.003 |


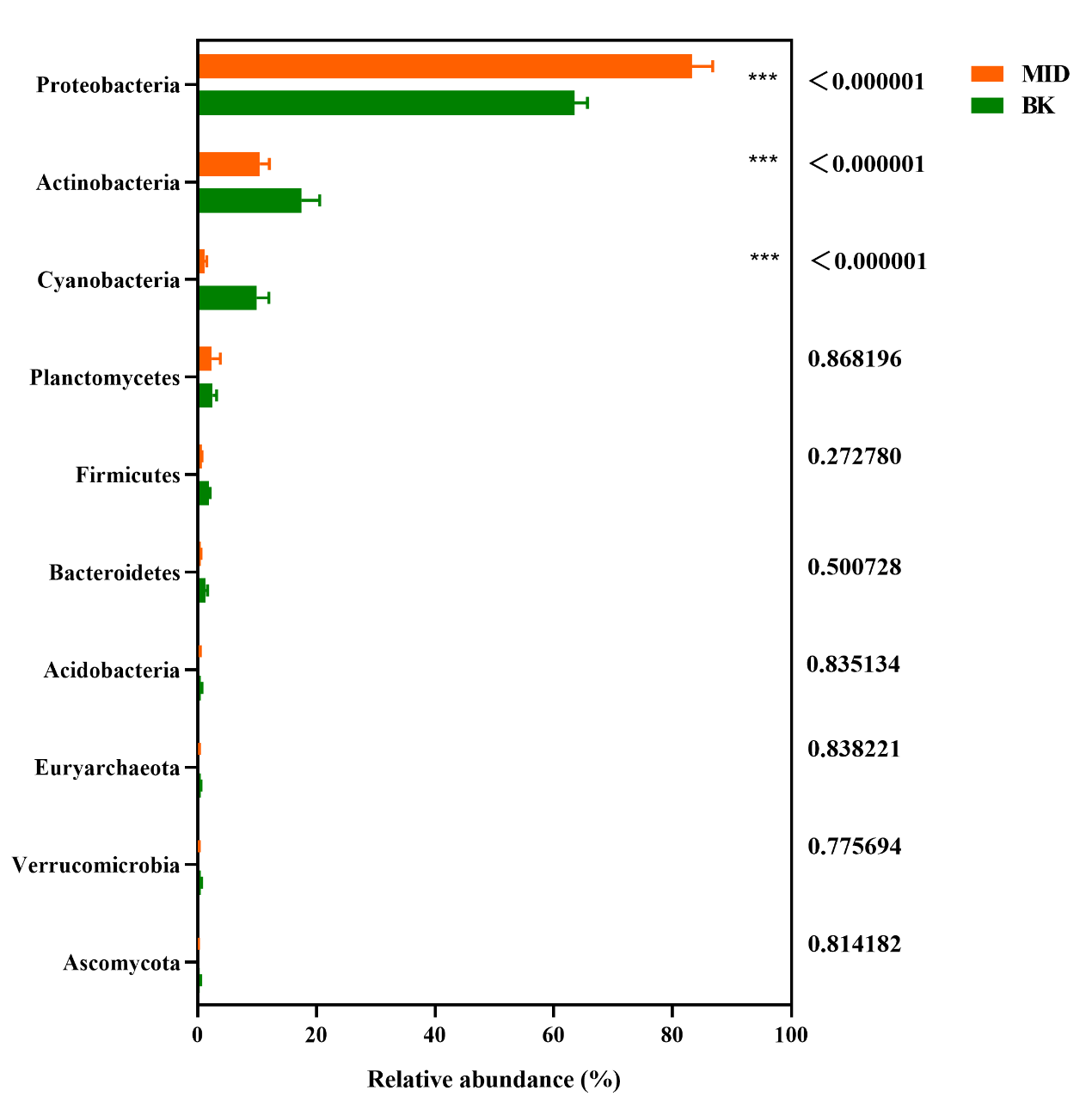


**Figure S1** Abundance difference of at phylum level between two communities

**Table S5** The abundance of the top 50 genera in different samples

| Phylum | Genus | Abundance of microbial genera | | | | | |
| --- | --- | --- | --- | --- | --- | --- | --- |
|  |  | MID | | | BK | | |
|  |  | Aug | Nov | Apr | Aug | Nov | Apr |
| Proteobacteria | *Lysobacter* | 15.670 | 13.613 | 15.320 | 0.620 | 1.029 | 0.932 |
|  | *Sphingomonas* | 1.763 | 2.205 | 1.944 | 3.473 | 8.116 | 7.853 |
|  | *Luteimonas* | 10.252 | 5.792 | 7.842 | 0.188 | 0.252 | 0.301 |
|  | *Pseudomonas* | 2.761 | 3.350 | 4.157 | 2.844 | 2.619 | 2.824 |
|  | *Stenotrophomonas* | 5.699 | 4.113 | 4.860 | 0.566 | 0.715 | 0.692 |
|  | *Xanthomonas* | 6.081 | 3.893 | 4.919 | 0.465 | 0.524 | 0.569 |
|  | *Pseudoxanthomonas* | 4.799 | 3.033 | 4.020 | 0.193 | 0.260 | 0.285 |
|  | *Mesorhizobium* | 2.023 | 2.012 | 2.150 | 1.916 | 2.358 | 1.913 |
|  | *Thermomonas* | 4.588 | 2.843 | 3.635 | 0.165 | 0.222 | 0.262 |
|  | *Bradyrhizobium* | 1.605 | 2.234 | 2.030 | 1.713 | 1.859 | 1.859 |
|  | *Sphingopyxis* | 1.422 | 2.066 | 1.896 | 1.184 | 2.531 | 2.001 |
|  | *Sphingobium* | 0.669 | 0.802 | 0.745 | 1.329 | 2.895 | 2.525 |
|  | *Burkholderia* | 1.291 | 1.345 | 1.320 | 1.725 | 1.205 | 1.464 |
|  | *Variovorax* | 0.729 | 0.888 | 0.955 | 1.887 | 0.762 | 1.195 |
|  | *Magnetospirillum* | 0.808 | 1.126 | 1.158 | 0.884 | 0.904 | 0.801 |
|  | *Rhizobium* | 0.690 | 0.911 | 0.889 | 0.963 | 1.091 | 0.899 |
|  | *Brevundimonas* | 0.222 | 0.267 | 0.297 | 3.067 | 0.793 | 0.514 |
|  | *Cupriavidus* | 0.722 | 0.785 | 0.771 | 1.032 | 0.709 | 0.834 |
|  | *Methylobacterium* | 0.498 | 0.627 | 0.599 | 0.738 | 0.625 | 0.755 |
|  | *Azospirillum* | 0.527 | 0.749 | 0.749 | 0.561 | 0.484 | 0.565 |
|  | *Terricaulis* | 0.181 | 0.157 | 0.330 | 0.762 | 1.464 | 0.693 |
|  | *Sorangium* | 0.219 | 0.237 | 0.204 | 1.411 | 0.753 | 0.682 |
|  | *Hydrogenophaga* | 0.395 | 0.886 | 0.390 | 1.025 | 0.353 | 0.430 |
|  | *Achromobacter* | 0.525 | 0.549 | 0.536 | 0.681 | 0.489 | 0.591 |
|  | *Salmonella* | 0.434 | 0.402 | 0.368 | 0.498 | 0.766 | 0.816 |
|  | *Acidovorax* | 0.292 | 0.320 | 0.295 | 1.316 | 0.462 | 0.593 |
|  | *Caulobacter* | 0.314 | 0.432 | 0.493 | 0.837 | 0.593 | 0.483 |
|  | *Paraburkholderia* | 0.434 | 0.517 | 0.470 | 0.647 | 0.492 | 0.575 |
|  | *Novosphingobium* | 0.245 | 0.307 | 0.298 | 0.501 | 0.974 | 0.789 |
|  | *Escherichia* | 0.113 | 0.121 | 0.107 | 2.088 | 0.256 | 0.166 |
|  | *Bordetella* | 0.467 | 0.436 | 0.443 | 0.560 | 0.375 | 0.455 |
|  | *Bosea* | 0.368 | 0.483 | 0.464 | 0.458 | 0.446 | 0.475 |
|  | *Massilia* | 0.427 | 0.427 | 0.420 | 0.556 | 0.370 | 0.442 |
|  | *Sinorhizobium* | 0.306 | 0.417 | 0.425 | 0.368 | 0.406 | 0.346 |
|  | *Erythrobacter* | 0.151 | 0.178 | 0.176 | 0.348 | 0.685 | 0.540 |
|  | *Hypericibacter* | 0.279 | 0.491 | 0.573 | 0.233 | 0.225 | 0.269 |
|  | *Paracoccus* | 0.304 | 0.339 | 0.356 | 0.375 | 0.348 | 0.342 |
|  | *Rhodopseudomonas* | 0.280 | 0.371 | 0.345 | 0.344 | 0.362 | 0.359 |
| Actinobacteria | *Streptomyces* | 2.639 | 2.778 | 2.460 | 6.366 | 3.491 | 5.242 |
|  | *Nocardioides* | 0.287 | 0.372 | 0.287 | 0.795 | 1.251 | 1.444 |
|  | *Pseudonocardia* | 0.373 | 1.128 | 0.238 | 0.474 | 0.449 | 1.353 |
|  | *Mycolicibacterium* | 0.476 | 0.551 | 0.469 | 0.632 | 0.531 | 0.704 |
|  | *Micromonospora* | 0.420 | 0.477 | 0.394 | 0.658 | 0.454 | 0.666 |
|  | *Mycobacterium* | 0.349 | 0.439 | 0.351 | 0.522 | 0.434 | 0.577 |
|  | *Microbacterium* | 0.297 | 0.361 | 0.305 | 0.578 | 0.465 | 0.565 |
|  | *Rhodococcus* | 0.273 | 0.309 | 0.255 | 0.393 | 0.341 | 0.443 |
| Cyanobacteria | *Chroococcidiopsis* | 0.023 | 0.023 | 0.012 | 1.179 | 5.771 | 4.407 |
|  | *Nostoc* | 0.205 | 0.191 | 0.117 | 0.921 | 1.002 | 1.033 |
|  | *Leptolyngbya* | 0.132 | 0.124 | 0.066 | 0.739 | 0.482 | 0.592 |
| Planctomycetes | *Lacipirellula* | 0.511 | 2.419 | 1.255 | 0.064 | 0.165 | 0.147 |
